# Urinary Neutrophil Gelatinase Associated Lipocalin: A Novel Biomarker Determining Steroid Responsiveness in Nephrotic Syndrome

**DOI:** 10.7759/cureus.34503

**Published:** 2023-02-01

**Authors:** Rakesh Kumar, Ravi Shekhar, Anand K Gupta, Amit Kumar, Nidhi Prasad, Santosh Kumar, Seema R Sinha, Jayant Prakash

**Affiliations:** 1 Pediatrics, Indira Gandhi Institute of Medical Sciences, Patna, IND; 2 Diabetes and Endocrinology, Indira Gandhi Institute of Medical Sciences, Patna, IND; 3 Community Medicine, Indira Gandhi Institute of Medical Sciences, Patna, IND; 4 Genetics, Indira Gandhi Institute of Medical Sciences, Patna, IND; 5 Hematology, Indira Gandhi Institute of Medical Sciences, Patna, IND

**Keywords:** kidney injury, protenuria, serum cholesterol, nephrotic syndrome, ngal

## Abstract

Background: Urinary Neutrophil Gelatinase Associated Lipocalin (uNGAL) has been demonstrated to be a powerful marker of progression in chronic kidney disease. The present study was done to find out the ability of uNGAL as a biomarker to differentiate steroid-sensitive nephrotic syndrome (SSNS), steroid-dependent nephrotic syndrome (SDNS), and steroid-resistant nephrotic syndrome (SRNS) from each other.

Method: The cross-sectional study included 45 patients with Idiopathic Nephrotic Syndrome (INS) (15 each of SSNS, SDNS, and SRNS). uNGAL was measured by ELISA. Demographic profile of patients with INS, lab parameters including Serum albumin, cholesterol, urinary albumin, creatinine, etc., were estimated using standard laboratory methods. Various statistical methods were used to assay the usefulness of NGAL as a diagnostic marker.

Results: Among the three groups, the median value of uNGAL was 8.68 ng/ml in SSNS, higher in SDNS (32.8 ng/ml), and highest in the SRNS group (50 ng/ml). The receiver operating curve (ROC) was generated for uNGAL to differentiate between SDNS and SSNS. Cut-off 13.26 ng/ml had a sensitivity of 86.7% and specificity of 97.4%, PPV 92.9%, and NPV 87.5 % with an area under the curve (AUC) of 0.958. Another ROC was generated for uNGAL to differentiate between SRNS and SDNS, and cut-off 40.02 ng/ml had a sensitivity of 80% and specificity of 86.7% with an AUC of 0.907. A similar result was observed when ROC was generated to differentiate SRNS from SSNS and SDNS combined.

Conclusion: uNGAL can distinguish between SSNS, SDNS, and SRNS.

## Introduction

Idiopathic nephrotic syndrome (INS) is a common glomerular disease in children, occurring in 16 per 100000 children [1]. The prognosis of children with INS depends largely on their response to steroid therapy. Based on the latter, INS is classified as a steroid-sensitive nephrotic syndrome (SSNS), steroid-dependent nephrotic syndrome (SDNS), and steroid-resistant nephrotic syndrome (SRNS). The initial clinical presentation of all these forms of INS is the same and includes edema, massive proteinuria, hypoalbuminemia, and hypercholesterolemia [2]. Unlike SSNS, children with SRNS do not show a response to steroid treatment. Because of this approach, children with SRNS are unnecessarily exposed to high doses of prolonged steroid therapy with no benefit. Such children often develop features of steroid toxicity [3,4].

Moreover, this approach also postpones alternative treatments that may have a better chance of success. Children with SDNS show a response to steroids initially, but the disease relapses when the child is put on alternate-day steroids or within two weeks of stopping the steroid. Now the child is again treated with a daily dose of steroid initially, and after achieving remission, a second drug is usually given with steroid on an alternate day basis. Early diagnosis of SDNS may help avoid prolonged steroid therapy and early initiation of 2nd line drugs. So, there is an urgent need for a marker to distinguish between SSNS, SDNS, and SRNS at the beginning. This will enable the treating pediatrician to make an appropriate decision, especially regarding the choice of the drug.

Urine has been said to be a non-invasive fluid biopsy of the kidney. Identifying urinary biomarkers that may predict steroid responsiveness will be highly beneficial in making an appropriate decision to manage INS. Timely addition of alternative drugs will lead to avoidance of exposure to high doses of prolonged steroid therapy.

Neutrophil gelatinase-associated lipocalin (NGAL) is a 25 kDa protein in various epithelial tissues. NGAL is predominantly expressed in the distal nephron segments of the kidney. NGAL promotes the epithelial differentiation of the mesenchymal progenitors during kidney development leading to the generation of glomeruli, proximal tubules, distal tubules, and loop of Henle [5]. Studies using animal models have shown NGAL as an early marker of renal injury [6,7]. Bennett et al. reported that urinary NGAL (uNGAL) had a high power to distinguish SRNS from SSNS [7]. In a recent study, uNGAL is a reliable marker of tubulointerstitial lesions in focal segmental glomerulosclerosis (FSGS) patients [8]. Also, NGAL has strongly predicted disease progression in patients with chronic kidney disease [9,10]. INS may present as SSNS, SDNS, or SRNS in the first episode. Sometimes, children with NS who were initially steroid responsive become steroid dependent later on and may become steroid resistant over time, depicting disease progression.

In the present study, we assessed the uNGAL as a potential urinary biomarker that can differentiate between SSNS, SDNS, and SRNS. Also, the variation of the level of uNGAL by age and correlation of the level of NGAL with urinary protein and creatinine and serum albumin and cholesterol was done.

## Materials and methods

This cross-sectional study was conducted on 45 patients of INS of age group 1-12 years who attended our pediatric nephrology clinic. Only those cases of INS who had been diagnosed as the first episode were included. The cases were subdivided into three groups- Group 1 consisted of Steroid Sensitive Nephrotic syndrome (SSNS) (15 in number), Group 2- Steroid Dependent Nephrotic syndrome (SDNS) (15 in number), and Group 3 - Steroid Resistant Nephrotic syndrome (SRNS) (15 in number). Based on the presence of generalized edema, heavy proteinuria (urine protein 3+ or 4+ by dipstick test or urine spot protein creatinine ratio > 2mg/mg in a morning urine specimen), and hypoalbuminemia (serum albumin <3 g/dL), the nephrotic syndrome was diagnosed. Patients were treated according to the Indian Society of Pediatric Nephrology recommendations [11]. Patients achieving remission (urine protein negative on dipstick or urine protein creatinine (Cr) ratio of <0.2mg/mg for three consecutive days) with prednisolone (60 mg/m2/day as a single oral dose) by 6 weeks of therapy were categorized as SSNS. Patients who did not achieve remission with this treatment were categorized as SRNS. Those cases who initially responded to steroid therapy, like cases of SSNS but relapsed when put on alternate-day steroid therapy or within 14 days of completion of steroid therapy were classified as SDNS.11 Relapse was defined as patients having proteinuria 2+ or more by dipstick or spot urine protein creatinine ratio of >2mg/mg for three consecutive days, previously in remission.

Exclusion criteria for cases included children with acute kidney injury, congenital nephrotic syndrome (onset of disease before 3 months of age), secondary causes of nephrotic syndrome (HIV nephropathy, Hepatitis B nephropathy, Lupus nephritis, Henoch Schonlein purpura), presence of urinary tract infection and those on nephrotoxic medications including cyclosporine and tacrolimus. Approval by Institute Ethical Committee was obtained, and informed consent was taken from the parents or legal representatives in all the cases.

Demographic and clinical profiles (urinalysis, serum Creatinine, history of steroid response, current remission or relapse status, pathology, and immunosuppressant or other drugs were obtained at the time of enrolment. Urine was collected and centrifuged for 5 min at 5000 ×g. The centrifuge was aliquoted and stored at −80°C. A maximum of two freeze-thaw cycles were permitted for use in the study. A fully automated clinical chemistry autoanalyzer was used to estimate urinary creatinine, and cholesterol and other lab parameters were also estimated. Urinary albumin was assayed using a nephelometer or dipstick. Urine NGAL was assayed using commercially available ELISA kits (human NGAL Elisa kit by Fine test, Wuhan, China) in the department of biochemistry.

Statistical analysis

All data collected were analyzed using SPSS version 16.0 software (SPSS Inc., Chicago, IL, USA). Kolmogorov Smirnov normality test was done to access the normality of the data of all parameters belonging to each group. The non-parametric data were analyzed using the Kruskal-Wallis test to compare groups. Mann-Whitney U tests were applied for post-hoc analysis, and proportions were compared using the Chi-square test. The receiver operating characteristic curve (ROC) was generated and used to determine the cut-off levels of uNGAL to distinguish between SDNS from SRNS and SDNS from SSNS. Karl Pearson correlation and Spearman’s rho correlation coefficients were calculated between uNGAL with other parameters. A two-tailed p-value of <0.05 was considered significant, and an appropriate critical limit was applied for post hoc analysis. Sensitivity, Specificity, Positive predictive value (PPV), Negative predictive value (NPV), and accuracy for the chosen cut-off from ROC were calculated.

## Results

**Figure 1:**
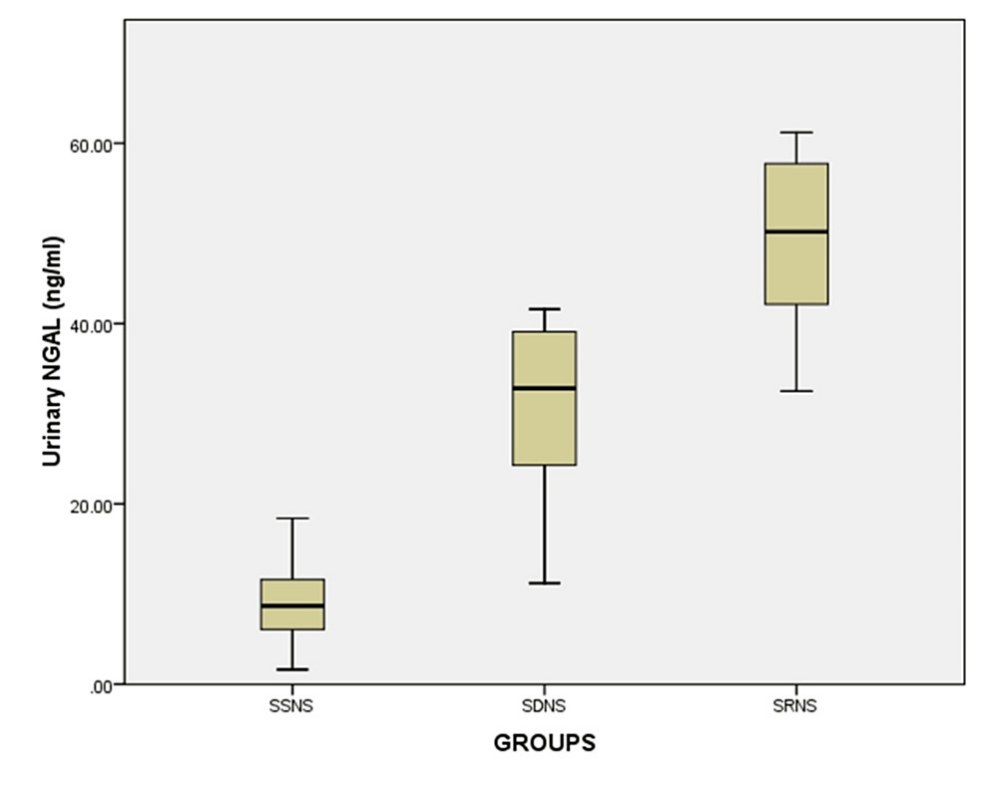
Comparison of uNGAL among various groups of Nephrotic syndrome based on response to steroids. uNGAL- Urinary Neutrophil Gelatinase Associated Lipocalin

The median values of uNGAL, serum albumin and serum cholesterol significantly differed among the SSNS, SDNS, and SRNS groups. Among these three groups, the median value of uNGAL was 8.68 ng/ml (IQR 4.97,11.65) in SSNS, higher in SDNS (32.8 ng/ml, IQR 23.4,39.6)) and highest in the SRNS group (50 ng/ml, IQR 40.1,58.9) (Figure 1).

There was a significant difference between the groups for urinary NGAL levels (H= 34.08, df=2, p < 0.001). Also, the median value of serum cholesterol was 328 mg/dl (IQR 310, 372) in SSNS, higher in SDNS (456 mg/dl, IQR 384,512) and highest in SRNS (620 mg/dl, IQR 596,636). There was an increasing trend of serum cholesterol from the SSNS to the SRNS group. The serum albumin was much lower in the SRNS groups (1.2 gm/dl. IQR 0.8-1.4) than in SDNS (1.7 gm/dl, IQR 1.2,2.0) and SSNS group (1.8 gm/dl, IQR 1.4,2.1). Urinary protein was highly elevated in the SRNS group (309 mg/dl, IQR 242,333) though there was a moderate difference between the SDNS (182 mg/dl, IQR 134,222) and SSNS (130 mg/dl IQR 84,279) group. The SRNS group had lower median urine creatinine compared to SDNS and SSNS. The urinary protein to creatinine ratio was high in SRNS, but there was a very poor difference between SDNS and SSNS for the ratio. There was no significant difference in the age and gender distribution, and they were comparable among these three groups. The basic characteristics are summarized (Table 1). 

**Table 1:** Demographic and Clinical data of studied patients a: Kruskal Wallis test; b: Chi-square test; c: Data are median with the inter-quartile range in parentheses SSNS: Steroid-sensitive nephrotic syndrome; SDNS: Steroid-dependent nephrotic syndrome; SRNS: Steroid-resistant nephrotic syndrome

VARIABLES	Group 1 SSNS (n=15)	Group 2 SDNS (n=15)	Group 3 SRNS (n=15)	Test Statistic	p Value
Age distribution at the time of study (years)	5 (4,6) ^c^	7 (4,9)^c^	8 (4,11) ^c^	4.046^a^	0.128^a^
Sex distribution, n (%) Male	9 (60)	10 (66.7)	9 (60)		
Female	6 (40)	5 (33.3)	6 (40)	0.189 ^b^	0.909 ^b^
Urinary NGAL (ng/ml)	8.68 (4.97,11.65) ^c^	32.8 (23.4,39.6) ^c^	50 (40.1,58.9) ^c^	34.087 ^a^	<0.001^a^
Serum albumin (g/dl)	1.8 (1.4,2.1) ^c^	1.7 (1.2,2.0) ^c^	1.2 (0.8,1.4) ^c^	9.733 ^a^	0.005 ^a^
Serum Cholesterol (mg/dl)	328 (310,372) ^c^	456 (384,512) ^c^	620 (596,636) ^c^	34.876 ^a^	<0.001^a^
Urinary Protein ( mg/dl)	130 (84,279) ^c^	182 (134,222) ^c^	309 (242,333) ^c^	18.362 ^a^	<0.001^a^
Urinary Creatinine(mg/dl)	73 (51,77.5) ^c^	72 (64,84) ^c^	54 (37,68) ^c^	3.803 ^a^	0.150 ^a^
Urinary protein creatinine ratio	2.31 (1.67,2.4) ^c^	2.94 (2.1,3.47) ^c^	5.37 (4,8.89) ^c^	20.870 ^a^	<0.001^a^

**Figure 2:**
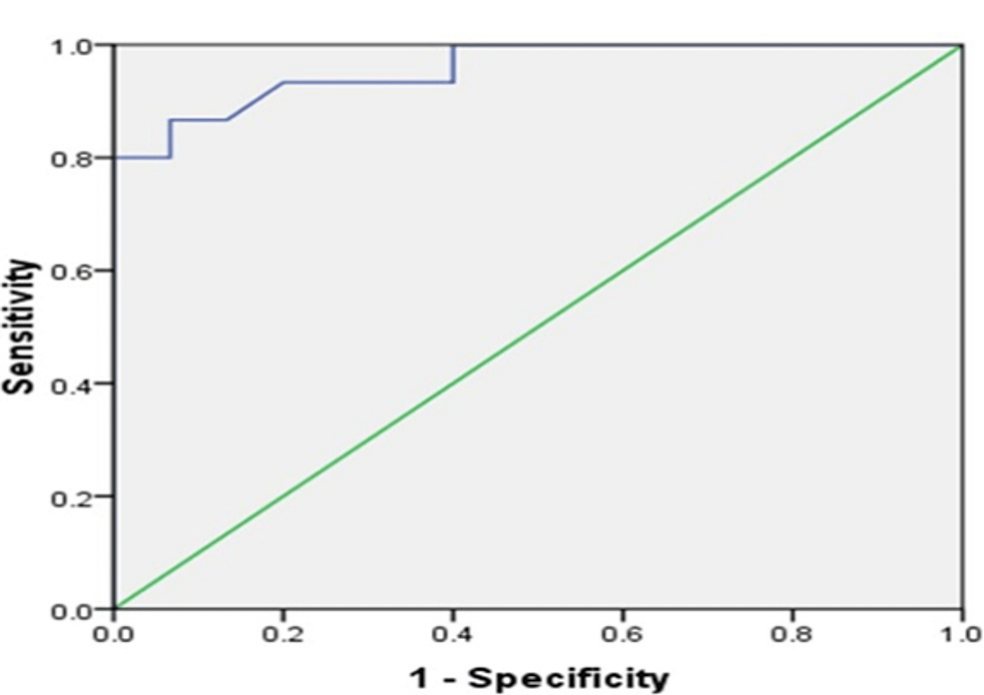
The receiver operating characteristic curve of uNGAL distinguishes SDNS from SSNS patients. SSNS: Steroid-sensitive nephrotic syndrome; SDNS: Steroid-dependent nephrotic syndrome; uNGAL: Urinary Neutrophil Gelatinase Associated Lipocalin

ROC was generated for uNGAL to differentiate between SDNS and SSNS cut-off 13.26 ng/ml had a sensitivity of 86.7% and specificity of 97.4%, PPV 92.9%, NPV 87.5 % with AUC of 0.958 (p<0.001,95% CI 0.893 - 1.023) (Figure 2).

**Figure 3:**
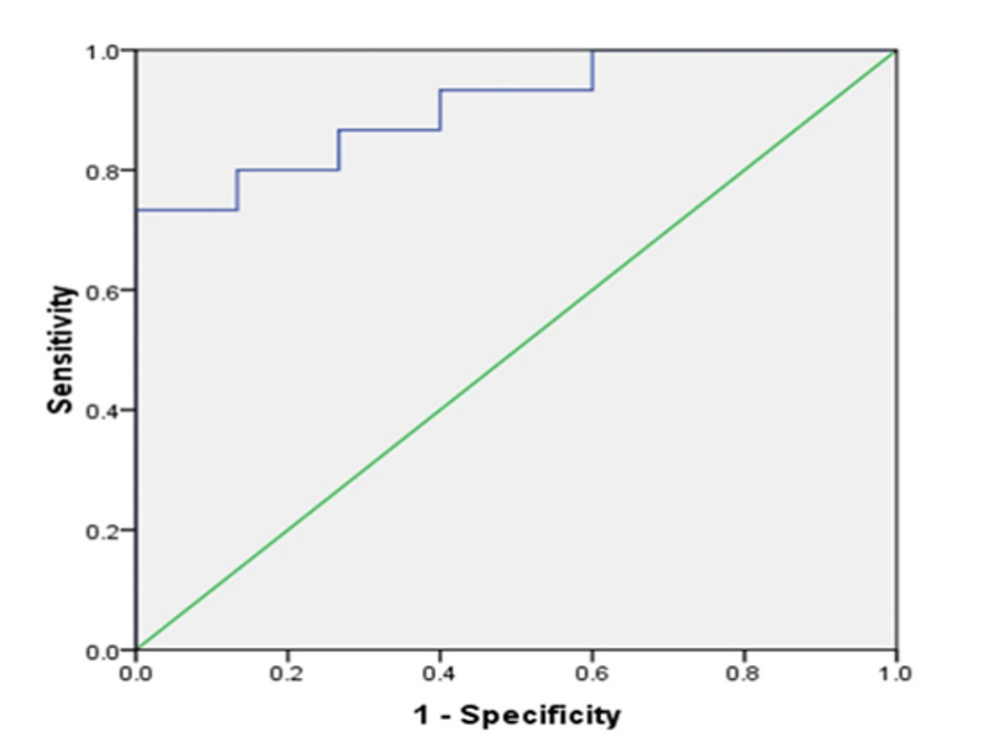
The receiver operating characteristic curve of uNGAL distinguishes SRNS from SDNS patients. SDNS: Steroid-dependent nephrotic syndrome; SRNS: Steroid-resistant nephrotic syndrome; uNGAL: Urinary Neutrophil Gelatinase Associated Lipocalin

**Figure 4:**
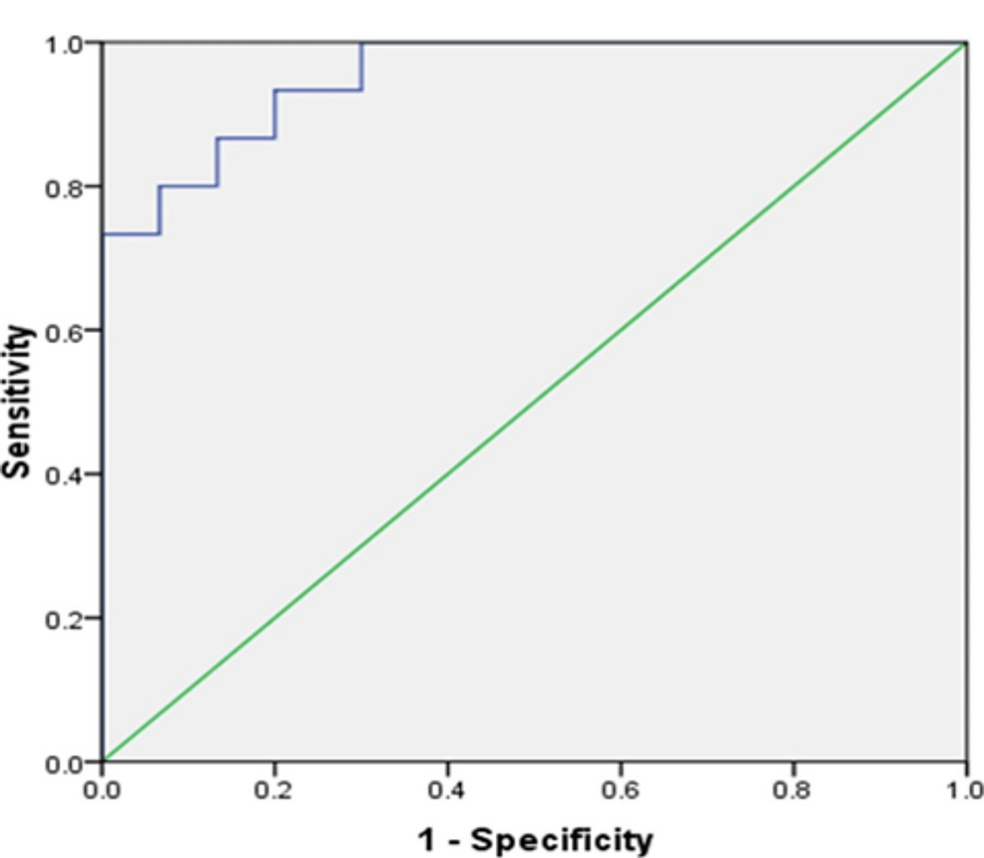
Receiver operating characteristic curve of uNGAL distinguishing of SRNS from SDNS and SSNS patients. SSNS: Steroid-sensitive nephrotic syndrome; SDNS: Steroid-dependent nephrotic syndrome; uNGAL: Urinary Neutrophil Gelatinase Associated Lipocalin; SRNS: Steroid-resistant nephrotic syndrome

Another ROC was generated for uNGAL to differentiate between SRNS and SDNS cut-off 40.02 ng/ml had a sensitivity of 80% and specificity of 86.7% with AUC of 0.907 (p<0.001,95% CI 0.8 - 1.013) (Figure 3). A similar result was observed when ROC was generated to differentiate SRNS from SSNS and SDNS combined (Figure 4). The AUC of the ROC mentioned above Curves, and their significance level with a 95% confidence limit is summarized (Table 2).

**Table 2:** Receiver operating characteristic (ROC) curves to determine the specificity and sensitivity of uNGAL to distinguish SDNS from other groups of Nephrotic Syndrome patients. AUC: area under the curve; PPV: positive predictive value; NPV: negative predictive value; SSNS: Steroid-sensitive nephrotic syndrome; SDNS: Steroid-dependent nephrotic syndrome; uNGAL: Urinary Neutrophil Gelatinase Associated Lipocalin; SRNS: Steroid-resistant nephrotic syndrome

	AUC	Cut Off	Sensitvity	Specificity	PPV	NPV	Accuracy
SDNS vs SSNS	0.958	13.26	86.70%	93.30%	92.90%	87.50%	90%
SRNS vs. SDNS	0.907	40.02	80.00%	86.70%	85.70%	81.20%	83.30%
SRNS vs. (SDNS+SSNS)	0.953	40.02	80.00%	93.30%	85.70%	90.30%	88.90%

The Spearman’s rho correlation coefficients of urinary NGAL with age, albumin, cholesterol, urinary creatinine, urinary protein, and urine protein/creatinine ratio in nephrotic syndrome patients were calculated (Table 3).

**Table 3:** uNGAL Pearson correlation coefficient and Spearman rank correlation with different variables uNGAL: Urinary Neutrophil Gelatinase Associated Lipocalin

Variables	Pearson Correlation	p Value	Spearman’s rho	p Value
Serum albumin	-0.533	<0.001	-0.577	<0.001
Age	0.25	0.09	0.334	0.025
Serum cholesterol	0.83	<0.001	0.834	<0.001
Urinary protein	0.57	<0.001	0.571	<0.001
Urinary creatinine	-0.93	0.542	-0.101	0.509
Urinary protein creatinine ratio	0.450	0.002	0.605	<0.001

**Figure 5:**
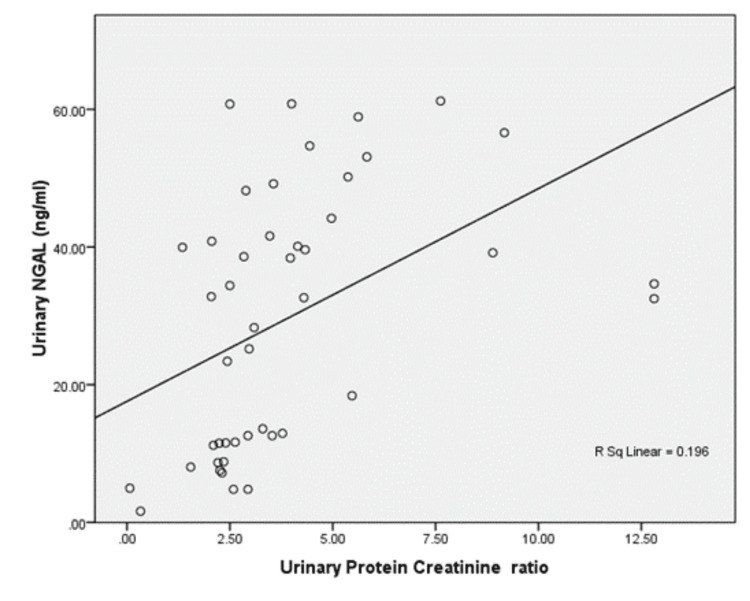
Correlation analysis of uNGAL with urinary protein creatinine ratio uNGAL: Urinary Neutrophil Gelatinase Associated Lipocalin

Urinary NGAL significantly negatively correlated with serum albumin (ρ= -0.538, p < 0.001). Urinary NGAL was found to have positive correlations with urinary protein (ρ= 0.571, p< 0.001) and serum cholesterol (Pearson r = 0.83, p <0.001). There was a positive correlation of uNGAL with urine protein creatinine ratio also (ρ=0.595, p<0.001) (figure 5).

## Discussion

Steroid-resistant nephrotic syndrome is associated with poor outcomes and gradual progression to end-stage renal disease (ESRD) [12]. A recent study on renal biopsy in cases of INS has shown a rising trend in cases of SRNS [13]. Steroid-dependent patients may have a prolonged course, especially when not diagnosed early [14]. In the long term, the risk of relapse and the adverse effects of the treatments remain the main concerns. No diagnostic marker is available currently to distinguish SSNS, SDNS, and SRNS from each other. NGAL can independently predict progression to CKD [9,10]. In this study, our objective was to determine if uNGAL measurements could distinguish SSNS, SDNS, and SRNS from each other so that appropriate drugs may be started leading to an early positive outcome. Early information about the nature of the disease will enable the treating pediatrician to prognosticate the apprehensive parents appropriately.

Our results showed a marked increase in levels of uNGAL in patients with SRNS versus SDNS and SSNS, and among the latter two, SDNS cases have higher uNGAL than SSNS cases. Other researchers have recently found the uNGAL level higher in SRNS than in SSNS [15-17]. However, we found no study done on uNGAL values in SDNS cases. Further, we found that at a cut-off level of 40.02 ng/dl, uNGAL had a sensitivity of 72.7% and specificity of 77.4% with AUC of 0.8 (p<0.001, 95% confidence interval 0.723-1.000) to differentiate patients of SRNS from SDNS. A similar cut-off with higher specificity and accuracy was generated when uNGAL was compared between SRNS as one group and SDNS and SSNS combined as another group. Other studies on SRNS and SSNS cases found lower cut-off values for differentiation among these two [15-17]. In our study, another cut-off of 13.02 ng/ml of NGAL was found to differentiate between SDNS and SSNS with a sensitivity of 86.7% and specificity of 97.4%, PPV of 92.9%, NPV of 87.5 % with AUC of 0.958 (p<0.001,95% CI 0.893 - 1.023). These results are consistent with our hypothesis that since SRNS is a disease with marked renal injury and having a high risk of progression to ESRD and earlier research showing uNGAL level to be higher in cases with worsening renal function, patients with SRNS would have higher levels of uNGAL [18-20]. SDNS has a better prognosis than SRNS [14]. Children with SDNS respond to steroid therapy but relapse when the steroid is stopped. SSNS cases usually have the most favorable outcome among the three. When we see the median value of uNGAL in all these three groups, we find a pronounced increasing trend in the level of uNGAL, with the lowest in SSNS and highest in SRNS. A recent study on humans and mice concluded that uNGAL might be useful in monitoring disease activity and treatment efficacy in various forms of renal injury [6]. We propose that uNGAL may have prognostic value. The increasing trend indicates the increasing severity of the disease.

INS is accompanied by disordered lipid metabolism. In our study, like uNGAL level, serum cholesterol level also showed an increasing trend with the progression of disease with lower values in SSNS, higher in SDNS, and higher in SRNS. In one recent study, serum cholesterol level was significantly elevated in SRNS compared to other types [21]. A recent study found that higher levels of serum cholesterol may be associated with increased severity and chronicity of kidney disease [22]. There is a positive correlation of uNGAL with urine protein, suggesting that high levels of albumin in urine are associated with increased levels of uNGAL. A correlation was found to be significant. Increased tubular damage is expected in SRNS compared to SDNS and SSNS, a progressive form of NS with poor prognosis, resulting in the excretion of low molecular proteins such as uNGAL in urine [18]. Previous studies have also found a significant positive correlation between uNGAL levels with proteinuria in patients with proteinuric CKD. Moreover, the uNGAL had significant positive correlations with serum cholesterol and negative correlations with serum albumin. There was no significant correlation between uNGAL with age.

One recent study used a panel of biomarkers, including NGAL, fetuin-A, vitamin D binding protein, alpha-1 acid glycoprotein 2, and prealbumin. The study found the use of biomarker panels useful [23]. However, testing many such markers in each patient may be quite cumbersome and will prove to be very costly. Some of the candidate urinary and serum biomarkers which have been researched and have potential for clinical application shortly include CD80, CD40, hemopexin, Soluble Urokinase Plasminogen Activator Receptor, Cardiotrophin- Like Cytokine Factor 1, Angiopoietin-Like Factor 4 and some of the genetic mutations which when diagnosed by either targeted sequencing of candidate genes or by whole genome sequencing may work as genetic markers of different NS [24].

Our study has limitations. This was a pilot study with a small sample size. Also, in many cases, the estimation of uNGAL was done on patients already on steroid treatment of varied duration. As of now, we do not know the effect of steroids on the urinary excretion of NGAL. So, a large prospective study that will determine uNGAL levels at baseline before the start of treatment and follow-up of uNGAL levels at regular intervals with the progress of the disease is required. Biopsy-proven diagnosis of all the cases is also required.

## Conclusions

We conclude that uNGAL can differentiate SRNS from SDNS and SDNS from SSNS. To the best of our knowledge, this is the only study that has been done comparing the level of uNGAL in all three subtypes SRNS, SDNS, and SSNS. Our study included only new cases (1st episode) of INS, thus avoiding the effect of the duration of the disease on the level of uNGAL. Thus uNGAL may be used to get valuable information based on which clinicians may individualize the treatment. Further studies are needed to measure uNGAL in steroid naïve cases of INS and follow-up of uNGAL levels at regular intervals.

## References

[1] Gipson DS, Massengill SF, Yao L (2009). Management of childhood onset nephrotic syndrome. Pediatrics.

[2] Eddy AA, Symons JM (2003). Nephrotic syndrome in childhood. Lancet.

[3] Trautmann A, Schnaidt S, Lipska-Ziętkiewicz BS (2017). Long-term outcome of steroid-resistant nephrotic syndrome in children. J Am Soc Nephrol.

[4] Agrwal S, Mantan M, Dabas A, Batra VV (2022). Childhood steroid-resistant nephrotic syndrome: long-term outcomes from a tertiary care center. Indian J Nephrol.

[5] Mishra J, Ma Q, Prada A (2003). Identification of neutrophil gelatinase-associated lipocalin as a novel early urinary biomarker for ischemic renal injury. J Am Soc Nephrol.

[6] Kuwabara T, Mori K, Mukoyama M (2009). Urinary neutrophil gelatinase-associated lipocalin levels reflect damage to glomeruli, proximal tubules, and distal nephrons. Kidney Int.

[7] Bennett MR, Piyaphanee N, Czech K, Mitsnefes M, Devarajan P (2012). NGAL distinguishes steroid sensitivity in idiopathic nephrotic syndrome. Pediatr Nephrol.

[8] Korzeniecka-Kozerska A, Wasilewska A, Tenderenda E, Sulik A, Cybulski K (2013). Urinary MMP-9/NGAL ratio as a potential marker of FSGS in nephrotic children. Dis Markers.

[9] Bolignano D, Lacquaniti A, Coppolino G (2009). Neutrophil gelatinase-associated lipocalin (NGAL) and progression of chronic kidney disease. Clin J Am Soc Nephrol.

[10] Nishida M, Kawakatsu H, Okumura Y, Hamaoka K (2010). Serum and urinary neutrophil gelatinase-associated lipocalin levels in children with chronic renal diseases. Pediatr Int.

[11] Sinha A, Bagga A, Banerjee S (2021). Steroid sensitive nephrotic syndrome: revised guidelines. Indian Pediatr.

[12] Roberti I, Vyas S (2010). Long-term outcome of children with steroid-resistant nephrotic syndrome treated with tacrolimus. Pediatr Nephrol.

[13] Gulati S, Sharma AP, Sharma RK, Gupta A (1999). Changing trends of histopathology in childhood nephrotic syndrome. Am J Kidney Dis.

[14] Niaudet P (2009). Long-term outcome of children with steroid-sensitive idiopathic nephrotic syndrome. Clin J Am Soc Nephrol.

[15] Elaziz El-Gamasy MA, Abdelhafez MA, Barr MM (2017). Urinary neutrophil gelatinase-associated lipocalin as prognostic biomarker for idiopathic nephrotic syndrome in Egyptian children. J Integr Nephrol Androl.

[16] Nickavar A, Safaeian B, Sadeghi-Bojd S, Lahouti Harah dashti A (2016). Urine neutrophil gelatinase associated lipocalin to creatinine ratio: a novel index for steroid response in idiopathic nephrotic syndrome. Indian J Pediatr.

[17] Choudhary A, Mohanraj PS, Krishnamurthy S, Rajappa M (2020). Association of urinary vitamin D binding protein and neutrophil gelatinase-associated lipocalin with steroid responsiveness in idiopathic nephrotic syndrome of childhood. Saudi J Kidney Dis Transpl.

[18] Coppolino G, Comi N, Bolignano D (2020). Urinary neutrophil gelatinase-associated lipocalin (NGAL) predicts renal function decline in patients with glomerular diseases. Front Cell Dev Biol.

[19] Bolignano D, Lacquaniti A, Coppolino G, Campo S, Arena A, Buemi M (2008). Neutrophil gelatinase-associated lipocalin reflects the severity of renal impairment in subjects affected by chronic kidney disease. Kidney Blood Press Res.

[20] Bolignano D, Coppolino G, Lacquaniti A, Nicocia G, Buemi M (2008). Pathological and prognostic value of urinary neutrophil gelatinase-associated lipocalin in macroproteinuric patients with worsening renal function. Kidney Blood Press Res.

[21] Krishanamurthy C, Rukmani J, Clarin D (2018). Evaluation of serum lipid profile in children with nephrotic syndrome admitted in emergency ward of Government Tirunelveli Medical College and Hospital, India. Int J Contemp Pediatr.

[22] Agrawal S, Zaritsky JJ, Fornoni A, Smoyer WE (2018). Dyslipidaemia in nephrotic syndrome: mechanisms and treatment. Nat Rev Nephrol.

[23] Bennett MR, Pleasant L, Haffner C (2017). A novel biomarker panel to identify steroid resistance in childhood idiopathic nephrotic syndrome. Biomark Insights.

[24] Stone H, Magella B, Bennett MR (2019). The search for biomarkers to aid in diagnosis, differentiation, and prognosis of childhood idiopathic nephrotic syndrome. Front Pediatr.

